# Protein Arginine Methyltransferases in γ-Globin Regulation and Sickle Cell Disease: Emerging Connections to Oxidative Stress

**DOI:** 10.3390/antiox15030324

**Published:** 2026-03-05

**Authors:** Waseem Chauhan, Rahima Zennadi

**Affiliations:** Department of Physiology, The University of Tennessee Health Science Center, 71 S. Manassas St., Memphis, TN 38163, USA

**Keywords:** oxidative stress, gamma-globin, protein arginine methyltransferases, reactive oxygen species, red blood cell, sickle cell disease

## Abstract

Reactive oxygen species (ROS) are unavoidable byproducts of cellular metabolism and are normally controlled by tightly regulated antioxidant systems. Red blood cells (RBCs) are particularly susceptible to oxidative stress due to their high oxygen exposure and iron content. In sickle cell disease (SCD), this vulnerability is exacerbated, as sickled RBCs generate chronically elevated ROS that contribute directly to disease pathophysiology. This review examines emerging evidence linking oxidative stress responses to regulation of fetal hemoglobin (HbF) expression through protein arginine methyltransferases (PRMTs). PRMTs catalyze arginine methylation of histone and non-histone substrates, thereby shaping chromatin structure, transcriptional programs, and translational control. We highlight recent findings demonstrating that specific PRMTs regulate γ-globin expression through distinct mechanisms, including transcriptional repression at the β-globin locus and post-transcriptional control of γ-globin mRNA translation. We propose that oxidative stress signaling may modulate PRMT activity, creating a mechanistic link between cellular stress responses and HbF induction. Because HbF inhibits pathological hemoglobin S polymerization, PRMT-dependent pathways represent an attractive therapeutic axis for SCD and related β-hemoglobinopathies. By integrating oxidative stress biology with PRMT-mediated epigenetic and translational regulation, this review outlines a unifying framework for HbF control, identifies critical knowledge gaps, and highlights future directions for the development of targeted epigenetic therapies.

## 1. Introduction

Under physiological conditions, intracellular catabolic processes that use oxygen as an electron acceptor generate reactive oxygen species (ROS). A tightly regulated balance between pro-oxidant and antioxidant systems normally prevents oxidative damage to cellular components. However, under pathological stress, this equilibrium is disrupted, triggering oxidative reactions that damage DNA, proteins, and membrane lipids and contribute to diverse disease processes [1,2].

Among circulating cells, red blood cells (RBCs) are uniquely vulnerable to oxidative stress due to their constant exposure to oxygen and high intracellular iron content. To counteract this, RBCs possess robust antioxidant defense systems. In sickle cell disease (SCD)—an autosomal recessive disorder caused by a point mutation in the β-globin gene that substitutes valine for glutamic acid at position six and results in production of sickle hemoglobin (HbS) [3]—this protective capacity is overwhelmed. In sickle RBCs, pathological ROS generation arises from HbS autoxidation and increased NADPH oxidase (NOX) activity, which are central drivers of SCD pathophysiology [4,5,6].

Under hypoxic conditions, HbS polymerization distorts RBC morphology from a flexible biconcave disc into a rigid sickle shape, promoting microvascular occlusion, ischemia–reperfusion injury, and recurrent vaso-occlusive pain crises. Chronic hemolysis, endothelial adhesion, and progressive organ damage ultimately reduce life expectancy [7,8], particularly among the approximately 100,000 individuals affected in the United States, most of whom are of African ancestry.

Despite advances in care, therapeutic options for SCD remain limited in efficacy and accessibility, underscoring the need for novel molecular targets. Several signaling pathways—including nuclear factor erythroid 2-related factor 2 (Nrf2)–Kelch-like ECH-associated protein 1 (Keap1), glutathione metabolism, mammalian target of rapamycin (mTOR), Akt, and protein arginine methyltransferases (PRMTs)—have been implicated in the regulation of oxidative stress responses [9,10,11].

PRMTs catalyze arginine methylation of histone and non-histone proteins and are increasingly recognized as key regulators of epigenetic signaling, cellular stress responses, and hematopoietic function [12]. Dysregulation of PRMT activity is linked to hematologic disorders, and emerging evidence suggests that PRMTs may contribute to oxidative injury in SCD while also influencing γ-globin expression, revealing potential therapeutic opportunities.

In this review, we systematically examine how PRMTs function as molecular sensors and effectors of oxidative stress in erythroid cells and how they regulate γ-globin expression through multilayered transcriptional, post-transcriptional, and signaling networks. By integrating insights from hematopoiesis, redox biology, and epigenetic regulation, we evaluate mechanistic links between ROS-mediated modulation of PRMT activity and fetal hemoglobin (HbF) induction, and assess the therapeutic potential of targeting specific PRMT isoforms in SCD and related β-hemoglobinopathies [13,14,15,16]. This framework highlights PRMT-centered redox regulation as a unifying concept and identifies new avenues for developing epigenetic, stress-responsive strategies for HbF induction.

## 2. The Role of γ-Globin in Hemoglobinopathies

Hemoglobinopathies are inherited disorders characterized by structural and/or developmental abnormalities in hemoglobin that result in significant clinical morbidity. In humans, hemoglobin synthesis follows a tightly regulated developmental program that begins in the embryonic stage, transitions through fetal life, and culminates in adulthood. These stages are defined by sequential globin gene expression and the production of distinct hemoglobin species, including embryonic hemoglobins [ζ_2_γ_2_ (Hb Portland-1), ζ_2_ε_2_ (Hb Gower-1), and α_2_ε_2_ (Hb Gower-2)], HbF, and adult hemoglobin (HbA), composed of two α and two β chains. Developmental globin regulation is governed by cis-acting regulatory elements, including the beta (β)-locus control region (β-LCR) within the β-globin gene cluster and the multispecies conserved sequence (MCS-R1–4) elements of the α-globin locus, together with stage-specific transcription factors and epigenetic modifiers [17].

In hemoglobinopathies, particularly SCD and β-thalassemia, sustained expression of γ-globin mitigates disease severity due to the higher oxygen affinity and anti-polymerization properties of HbF relative to HbA [18]. However, shortly after birth, a developmental hemoglobin switch represses γ-globin expression and activates β-globin transcription, resulting in predominance of HbA [19]. In SCD and β-thalassemia, pathogenic mutations in the β-globin gene lead to heterogeneous but overlapping clinical manifestations, including HbS polymerization and vaso-occlusive pathology in SCD, excess free α-globin chains in β-thalassemia, increased reactive oxygen species (ROS), extramedullary erythropoiesis, and ineffective erythropoiesis [20].

As a result, therapeutic efforts have focused on re-inducing γ-globin expression, which is physiologically silenced after birth. Experimental and clinical studies demonstrate that γ-globin reactivation reduces oxidative stress, decreases the frequency of vaso-occlusive and pain crises, and improves erythropoietic efficiency [21]. Accordingly, strategies that promote HbF induction—particularly those that intersect with oxidative stress and erythroid signaling pathways—represent a promising therapeutic avenue for the treatment of SCD and β-thalassemia.

## 3. How Is Oxidative Stress Involved in SCD?

In microcirculation, red blood cells (RBCs) normally neutralize exogenous reactive oxygen species (ROS) produced by neutrophils and macrophages, owing to their robust antioxidant capacity. This defense system includes non-enzymatic antioxidants such as glutathione (GSH) and ascorbic acid, as well as enzymatic antioxidants including superoxide dismutase (SOD), catalase, glutathione peroxidase (GPx), and peroxiredoxin-2 (PRDX2) [22].

In SCD, however, RBCs themselves become a major source of oxidative stress. This shift is driven primarily by hemoglobin S (HbS) instability and polymerization, as well as increased activity of NADPH oxidase (NOX) enzymes [4]. HbS undergoes enhanced autoxidation, generating excessive intracellular ROS that disrupt erythrocyte physiology [22,23]. While oxygenated hemoglobin can autoxidize under physiological conditions, HbS produces significantly more ROS than HbA in the presence of oxygen [23,24,25]. Repeated cycles of sickling and unsickling further amplify ROS generation. Because cytoplasmic antioxidants have limited access to the plasma membrane, ROS readily attack membrane lipids and proteins, resulting in cumulative oxidative damage [22,26,27]. In addition to HbS autoxidation, sickle RBCs generate ROS through Ca^2+^-, PKC-, and Rac-GTPase-regulated NOX enzymes [4,5,6,28]. NOX-derived ROS enhance endothelial adhesion, increase RBC fragility and hemolysis, and promote vaso-occlusion [5,28]. Impaired mitochondrial clearance further contributes to elevated ROS levels [29].

Sickle RBCs also exhibit compromised antioxidant defenses, with reduced levels of SOD, catalase, and PRDX2 [30,31], as well as decreased glutathione disulfide (GSSG), reduced GSH, and total GSH compared with healthy controls [32]. This imbalance between ROS production and antioxidant capacity disrupts redox homeostasis [6]. Persistent HbS oxidation and NOX activation lead to ROS accumulation that overwhelms membrane-associated and cytosolic antioxidants such as PRDX2, GSH, and GPx [5,31,32,33]. PRDX2 plays a key role in stabilizing HbS and limiting oxidative hemolysis, while thioredoxin (Trx) and GSH are essential for maintaining erythrocyte redox balance [31,34,35].

Because mature RBCs are enucleated and lack protein synthesis machinery, sickle RBCs are particularly vulnerable to oxidative damage [36]. Their antioxidant systems are only partially effective against sustained endogenous ROS, allowing unneutralized oxidants to compromise membrane integrity and cellular function [37,38]. Although damaged RBCs are normally cleared by macrophages [39,40], phagocytic clearance is impaired in SCD [41], leading to accumulation of apoptotic RBCs, heme-laden vesicles, and free hemoglobin and heme in the circulation. Oxidative injury further alters the hemorheological properties of sickle RBCs and stimulates additional ROS production by macrophages, endothelial cells, and neutrophils, thereby amplifying vascular and inflammatory damage [42,43].

## 4. Proteins Involved in γ-Globin Expression

The β-globin gene cluster on chromosome 11 comprises embryonic (ε), fetal (γG and γA), and adult (δ and β) globin genes that are expressed sequentially during development [44,45]. Upstream of this cluster lies the locus control region (LCR), a set of erythroid-specific cis-regulatory elements that enhance globin gene expression through long-range interactions with transcription factors and chromatin-modifying complexes [46,47,48]. Knockout and transgenic β-globin mouse models have been instrumental in defining principles of developmental gene regulation, gene competition, and distal enhancer activity [49,50]. Studies of the β-LCR have also provided foundational insights into long-distance chromosomal interactions, including its dynamic proximity to active promoters and engagement with broader chromatin architecture during erythroid maturation [44].

The fetal (γ)-to-adult (β) globin switch is of major therapeutic interest, as persistence or reactivation of fetal hemoglobin (HbF) can markedly ameliorate disease severity in β-thalassemia and sickle cell disease (SCD) [17,21,51]. Over the past three decades, extensive research has sought to define the molecular basis of HbF silencing and HbA activation [52]. Proposed mechanisms include chromatin looping, promoter competition, gene silencing, and transcriptional tracking [51,53]. Despite the identification of numerous regulatory proteins, the full regulatory logic governing globin switching remains incompletely understood [17,19,51,54,55,56]. Notably, recent genetic studies in human populations have provided important new insights into this complex process [57]. In erythroid cells, the β-LCR undergoes conformational changes that bring it into close proximity with globin gene promoters [58,59], facilitating recruitment of transcriptional regulators including MYB, GATA1, LIM domain-binding protein 1 (LDB1), Friend of GATA1 (FOG1), NF-E2, KLF1, BCL11A, the DRED complex, FOP1, and stem cell leukemia (SCL) factor [17].

Genome-wide association studies (GWAS) identified BCL11A on chromosome 2 as a major HbF-associated locus [60]. This zinc finger transcription factor is now recognized as a central repressor of γ-globin expression in adult erythroid cells [61]. Genetic depletion of BCL11A induces robust γ-globin expression in adult erythroid precursors [62,63]. Mechanistically, BCL11A recruits multiprotein repressor complexes—including GATA1, nucleosome remodeling and deacetylase (NuRD), FOG1, and SOX6—to the β-globin locus, establishing it as a genetically validated regulator of hemoglobin switching [29,30].

Kruppel-like factor 1 (KLF1/EKLF), an erythroid-specific zinc finger transcription factor that binds CACCC motifs, is essential for erythropoiesis and globin gene regulation. KLF1 directly activates β-globin transcription and indirectly promotes γ-globin repression through induction of BCL11A [64]. Deletion of Klf1 in mice results in lethal anemia due to β-globin deficiency [65,66], while mutations in the β-globin CACCC box disrupt KLF1 binding and cause β-thalassemia [55,56,57], often resulting in embryonic lethality between E14 and E15 [67,68,69]. Thus, KLF1 enforces the γ-to-β switch through both direct and indirect mechanisms. In contrast, the oncofetal RNA-binding protein LIN28B enhances γ-globin expression by inhibiting BCL11A translation [70,71,72].

SOX6, a high-mobility group (HMG)-box transcription factor, also contributes to globin regulation. In Sox6-deficient mice, embryonic β-like globins persist beyond their normal developmental window, and SOX6 represses εy-globin expression in murine models [73,74,75]. SOX6 further supports definitive erythropoiesis by promoting erythroid survival and maturation [76]. Although its precise role in human globin regulation is less well defined, SOX6 expression levels correlate inversely with γ-globin expression in human erythroid precursors [77]. Depending on cellular context, SOX6 can function as either a transcriptional activator or repressor [78,79] and cooperates with GATA1 and BCL11A to silence γ-globin in adult erythroid cells [44].

MYB regulates γ-globin expression through multiple pathways, including activation of BCL11A and ZBTB7A [80,81,82,83]. ATF4, identified through RNA-sequencing analyses of isogenic erythroid precursors, binds the HBS1L-MYB enhancer to control MYB expression [80,84]. Reduced β-globin synthesis leads to decreased ATF4 levels, resulting in diminished MYB and BCL11A expression [80]. ATF4 thus functions as a key mediator of stress-responsive globin regulation, linking erythroid stress signaling to HbF induction.

Friend of PRMT1 (FOP), a recently identified chromatin-associated factor, represents an emerging regulator of γ-globin repression. Knockdown of FOP induces robust HbF expression, including in β-thalassemic erythroid cells, suggesting potential therapeutic relevance [85]. FOP is methylated by PRMT1 and PRMT5 [86], implicating arginine methylation in chromatin-based globin gene regulation [86,87]. Although the precise molecular mechanisms remain to be defined, FOP appears to be a critical PRMT-responsive node within the globin regulatory network.

## 5. PRMTs as Epigenetic Regulators: Lessons from Oncology and Relevance to Hemoglobin Switching

PRMTs are a family of enzymes that catalyze the transfer of methyl groups to arginine residues on target proteins, thereby regulating a wide range of cellular processes, including chromatin remodeling, RNA processing, transcriptional control, and intracellular signaling. Histones represent prominent PRMT substrates, and arginine methylation of histone tails contributes to shaping the epigenetic landscape by modulating chromatin accessibility, nucleosome stability, and recruitment of transcriptional cofactors. These histone modifications influence gene expression both by altering histone–DNA interactions and by creating docking sites for effector proteins that recognize methylated arginine residues and propagate downstream transcriptional programs [88,89,90].

In mammals, eleven PRMTs have been identified and are classified primarily according to the type of arginine dimethylation they catalyze. Type I PRMTs (PRMT1, PRMT2, PRMT3, PRMT4/CARM1, PRMT6, and PRMT8) generate asymmetric dimethylarginine (ADMA), a modification generally associated with transcriptional activation, whereas type II PRMTs (PRMT5 and PRMT9) catalyze symmetric dimethylarginine (SDMA), which is more frequently linked to transcriptional repression. PRMT7 predominantly catalyzes monomethylation and is sometimes classified as a type III enzyme [88,89,90]. PRMT10 and PRMT11 have been identified based on sequence homology, although their enzymatic activity and biological roles remain incompletely defined. Dysregulation of PRMT activity disrupts normal transcriptional programs and has been implicated in developmental abnormalities, cancer, and hematologic disease.

Investigation of PRMTs in γ-globin gene regulation has been informed substantially by extensive work in oncology, where these enzymes are recognized as master regulators of epigenetic gene expression programs. In cancer, PRMTs—particularly PRMT1, PRMT4 (CARM1), PRMT5, and PRMT7—modulate cell fate, proliferation, and survival by depositing methyl marks on histones that alter chromatin architecture and transcription factor accessibility [91,92]. This paradigm provides a compelling framework for understanding how PRMTs might similarly regulate the fetal-to-adult hemoglobin switch.

A central mechanism involves symmetric dimethylarginine (SDMA) marks deposited by PRMT5. In malignant cells, PRMT5-mediated methylation of histone H4 arginine 3 (H4R3me2s) and histone H3 arginine 8 (H3R8me2s) is associated with transcriptional repression of tumor suppressor genes [93,94]. By analogy, PRMT5 activity has been proposed to contribute to developmental silencing of the γ-globin genes (HBG1/HBG2) through establishment of a repressive chromatin environment within the β-globin locus. In contrast, PRMT1 and CARM1 catalyze ADMA marks associated with transcriptional activation (e.g., H4R3me2a, H3R17me2a) and have been hypothesized to promote γ-globin expression. The balance between these opposing PRMT activities may therefore represent a critical, yet underexplored, regulatory layer governing hemoglobin switching.

Beyond chromatin regulation, PRMT function is closely linked to cellular stress responses, a central feature of SCD pathophysiology. Recent studies demonstrate that PRMT7 is dynamically recruited to chromatin and modulates transcription of stress-responsive genes [95,96,97]. In the context of SCD—where erythroid progenitors and mature red blood cells are subjected to chronic oxidative stress—it is conceivable that PRMT7 and other PRMTs become activated or dysregulated, resulting in adaptive epigenetic reprogramming. Such reprogramming may secondarily promote γ-globin reactivation as part of a broader protective response to oxidative and metabolic stress.

### 5.1. PRMT5 as a Central Epigenetic Repressor of γ-Globin

**Figure 1:**
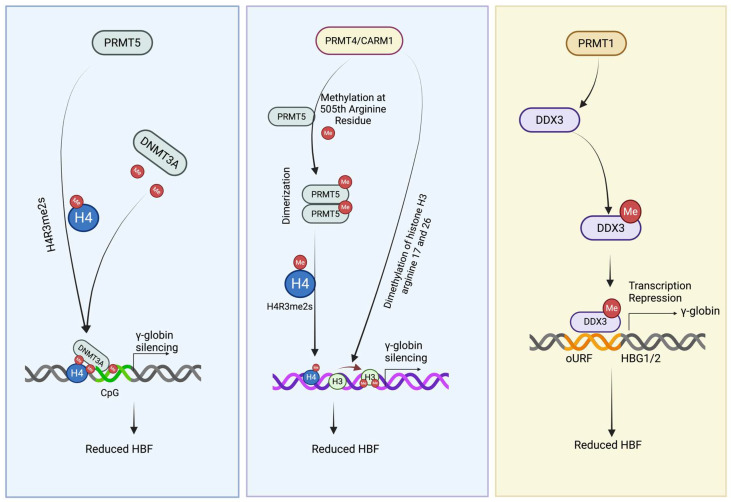
**Proposed mechanisms by which PRMT family members contribute to γ-globin silencing and reduced HbF expression**. (**Left panel**) PRMT5 catalyzes symmetric dimethylation of histone H4 at arginine 3 (H4R3me2s), creating a repressive chromatin mark. This modification facilitates recruitment of DNMT3A, which methylates CpG residues at the γ-globin promoter, leading to transcriptional repression and decreased HBF. (**Center panel**) PRMT4/CARM1 methylates PRMT5 on a specific arginine residue (S505), promoting PRMT5 dimerization and enhancing its enzymatic activity. The resulting increase in H4R3me2s and associated repressive histone modifications (e.g., H3 and H4 marks) strengthens γ-globin silencing, further reducing HBF levels. (**Right panel**) PRMT1 methylates the RNA helicase DDX3, which reduces its ability to facilitate ribosomal bypass of the upstream open reading frame (uORF) in the γ-globin (HBG1/2) 5′UTR. This methylation-dependent inhibition suppresses γ-globin translation without altering γ-globin mRNA abundance, thereby contributing to reduced HbF levels.

PRMT5 mediates symmetric dimethylation of histone H4 arginine 3 (H4R3me2s), a transcriptionally repressive chromatin mark enriched at multiple developmentally silenced loci, including the γ-globin genes (HBG1/HBG2) [86,98]. Genetic depletion or pharmacologic inhibition of PRMT5 reduces H4R3me2s deposition at the γ-globin promoter and results in reactivation of HbF expression, establishing PRMT5 as a key suppressor of γ-globin transcription (Figure 1, left panel) and a potential therapeutic target for hemoglobinopathies [86,98].

In addition to H4R3 methylation, PRMT5 symmetrically dimethylates arginine residues on histones H2A and H4, promoting chromatin compaction and transcriptional repression [99]. Beyond its role in chromatin regulation, PRMT5 is essential for embryonic stem cell pluripotency, hematopoietic stem and progenitor cell maintenance, and lineage-specific gene regulation during erythropoiesis, including globin gene switching [98,100]. PRMT5 also methylates a broad range of nonhistone substrates involved in RNA splicing, ribosome biogenesis, Golgi organization, and cell-cycle progression, underscoring its pleiotropic role in maintaining cellular homeostasis [88,99]. In erythroid cells, PRMT5 functions within repressive multiprotein complexes at the β-globin locus, where it cooperates with established γ-globin repressors and chromatin remodelers to enforce developmental silencing [86,98,101].

### 5.2. PRMT4/CARM1 Modulates PRMT5-Dependent Repression of γ-Globin

PRMT4, also known as coactivator-associated arginine methyltransferase 1 (CARM1), was initially identified through its interaction with GRIP1, a member of the p160 steroid receptor coactivator (SRC) family [102]. The SRC complex includes histone acetyltransferases such as CBP and p300, as well as arginine methyltransferases including CARM1 and PRMT1. These enzymes are recruited to gene promoters through nuclear receptor and transcription factor interactions, where they coordinate chromatin remodeling and transcriptional activation [103]. CARM1 primarily catalyzes asymmetric dimethylation of histone H3 at arginine 17 (H3R17me2a) and arginine 26 (H3R26me2a), modifications generally associated with transcriptional activation, and also methylates numerous nonhistone substrates involved in transcription, RNA splicing, and DNA damage responses [88].

In the context of γ-globin regulation, CARM1 plays an indirect but critical repressive role by modulating PRMT5 activity. CARM1 methylates PRMT5 at arginine 505, a modification that enhances PRMT5 dimerization and enzymatic activity, thereby promoting H4R3me2s deposition at the γ-globin promoter in K562 cells [101]. This cross-regulatory interaction reveals a hierarchical PRMT network in which a type I PRMT fine-tunes the repressive function of a type II PRMT to reinforce γ-globin silencing. Disruption of this regulatory axis reduces PRMT5 chromatin occupancy and relieves repression of γ-globin transcription (Figure 1, center panel) [101].

### 5.3. PRMT1 Regulates Post-Transcriptionally γ-Globin

Recent work has identified PRMT1, the predominant type I PRMT in mammalian cells, as a regulator of γ-globin expression acting post-transcriptionally, specifically at the level of mRNA translation [104]. In erythroid cells, genetic depletion or pharmacologic inhibition of PRMT1 results in a robust increase in γ-globin protein abundance without a corresponding increase in γ-globin mRNA levels [101], demonstrating that PRMT1 represses γ-globin expression independently of transcriptional control [101,104]. This dissociation between transcript and protein output highlights translational regulation as an important and previously underappreciated mechanism governing HbF expression.

Mechanistically, γ-globin mRNA contains a single non-AUG-initiated upstream open reading frame (uORF) within its 5′ untranslated region (UTR) that suppresses translation of the main coding sequence. Activation of the integrated stress response (ISR) promotes γ-globin translation despite this inhibitory uORF, although prolonged stress can ultimately reduce γ-globin mRNA abundance. In contrast to canonical ISR targets such as ATF4, which harbor multiple AUG-initiated uORFs, the unique uORF architecture of γ-globin suggests involvement of additional translational regulators beyond eIF2α signaling. One such regulator is the RNA helicase DDX3, which facilitates bypass of the γ-globin uORF by promoting initiation at near-cognate start codons within structured 5′UTRs [104,105]. PRMT1-mediated methylation of DDX3 suppresses this bypass mechanism, thereby repressing γ-globin translation (Table 1 below) without inducing overt cellular stress (Figure 1, right panel).

**Table 1:** Signaling Pathways Regulating PRMT1 and PRMT5 Activity Involved in Globin Expression and Hematopoiesis.

Stimulus	Signaling	Study	Reference
PRMT5 depletion	Increased mTOR signaling	Hematopoietic stem cells	[106]
Knockdown of FOP	Induces HbF, elevation of γ-globin expression through PRMT1 and PRMT5 arginine methyltransferases	β-thalassemic patients	[107]
PRMT1	Methylation of DDX3, suppress the cap dependent translation of γ-globin mRNA, engagement of a non-AUG uORF		[108]
Downregulation of ISR	Increase the translational efficiency of β-globin and γ-globin		[109]
PRMT5 deletion	Impaired cytokine signaling and increased p53 signaling	Steady-state adult hematopoiesis	[100]
PRMT5	Increase in p53 protein, Cdkn1a, Bbc3, Mdm2	HSPCs	[100]
PRMT5 loss	Impaired ERK1/2 phosphorylation	Normal primary hematopoietic cells	[110]
PRMT5 loss	Impaired cytokine driven STAT5 and AKT signaling	Hematopoietic cells	[110]
PRMT5 deletion	Targets spliceosome components, impair both constitutive splicing and alternative splicing	HSC	[111]

More broadly, multiple components of the translational machinery are regulated by arginine methylation, and several PRMT family members have been shown to influence protein synthesis through distinct mechanisms [112,113,114]. In this context, PRMT1-mediated control of γ-globin translation adds an important post-transcriptional layer to fetal hemoglobin (HbF) regulation. When considered alongside prior evidence that PRMTs also regulate γ-globin transcription, these findings suggest that targeting PRMT activity may enable a dual transcriptional and translational strategy for therapeutic HbF induction in hemoglobinopathies [104].

Collectively, these studies establish PRMT1 as a non-canonical regulator of HbF that is mechanistically distinct from classical chromatin-based repressors controlling γ-globin transcription. By acting at the level of translation, PRMT1 expands the regulatory landscape governing globin gene expression and provides a mechanistic explanation for the frequently observed discordance between γ-globin mRNA and protein levels in erythroid cells. Targeting PRMT1 therefore represents a novel therapeutic avenue for HbF induction that may complement existing transcription-focused approaches.

## 6. Why Are PRMTs Important for Normal Hematopoiesis?

Epigenetic regulators are essential for normal hematopoiesis, and their dysregulation is strongly implicated in hematologic malignancies [98]. PRMT1 is required for healthy hematopoiesis, as its deficiency in animal models leads to reduced hematopoietic stem cell (HSC) numbers and impaired erythroid, myeloid, and lymphoid development [115]. PRMT1 regulates HSC differentiation and self-renewal by modulating key transcription factors and signaling pathways, and its loss can promote myeloid transformation, a malignant disorder of blood-forming cells [115,116]. One critical substrate of PRMT1 is the transcription factor RUNX1, which plays an essential role in myeloid differentiation and lymphocyte development; PRMT1-mediated methylation of RUNX1 is required for proper hematopoietic lineage commitment [117]. Collectively, these findings establish PRMT1 as a central epigenetic regulator of hematopoiesis [115].

PRMT5, another key arginine methyltransferase, is highly expressed in HSCs and is indispensable for adult hematopoiesis. Conditional deletion of Prmt5 in mice results in severe pancytopenia caused by depletion of the HSC pool, impaired blood cell production, and disruption of cytokine and p53 signaling pathways [100]. This defect rapidly compromises hematopoietic output and can produce a phenotype resembling severe aplastic anemia. PRMT5 regulates gene expression by methylating transcription factors and chromatin-associated proteins [100] and represses multiple cell-cycle regulators, including cyclin E1, SMAD7, CDKN2A, and RB [118,119]. In addition, PRMT5 controls cell-cycle progression through methylation of nonhistone substrates such as FEN1, p53, and E2F1 [120,121]. In PRMT5-deficient hematopoietic stem and progenitor cells (HSPCs), activation of p53 and its downstream targets (e.g., Cdkn1a, Mdm2, Bbc3) leads to elevated p21 expression and cell-cycle arrest (Table 1) [122,123]. Together, these data demonstrate that PRMT5 is essential for HSC maintenance and function, underscoring the need for careful therapeutic targeting of PRMT5-dependent pathways in hematopoietic disease.

## 7. The Potential Interplay of Oxidative Stress and PRMT Activity in γ-Globin Regulation in SCD

PRMTs are well-recognized regulators of post-translational arginine methylation and influence a wide range of cellular functions, including epigenetic regulation, signal transduction, and cell-cycle control. Emerging evidence also implicates PRMTs in mitochondrial biology, particularly through their roles in maintaining intracellular calcium homeostasis [124], suggesting that specific PRMT isoforms may contribute to mitochondrial function and stress responses.

Mitochondria are the primary endogenous source of ROS, generating an estimated 0.2–2% of the oxygen consumed by the cell. Under physiological conditions, mitochondrial quality-control mechanisms—including antioxidant enzymes and mitophagy—limit ROS accumulation and preserve organelle integrity [125]. However, in SCD, a subset of circulating RBCs abnormally retain mitochondria, a feature not characteristic of healthy mature RBCs. These mitochondria-retaining RBCs exhibit markedly elevated ROS levels [29,126], indicating that persistent mitochondrial retention contributes substantially to oxidative stress in SCD. Consequently, mitochondrial dysfunction and impaired clearance become major drivers of intracellular ROS production in sickled erythrocytes.

### 7.1. PRMTs and Akt/PI3K & mTOR Signaling

**Figure 2:**
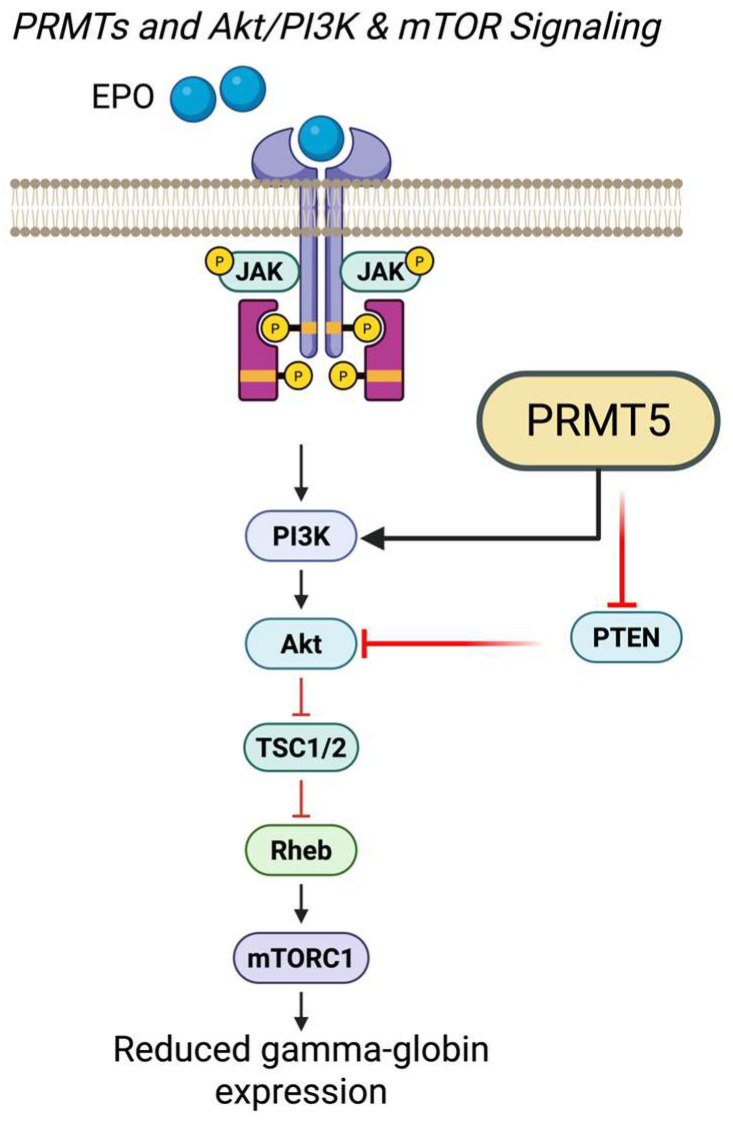
PRMT5 integration with erythropoietin-dependent PI3K/AKT/mTOR signaling and regulation of γ-globin expression. Schematic illustrating how PRMTs, with emphasis on PRMT5, intersect with EPO receptor signaling to modulate the PI3K/AKT/mTOR pathway in erythroid cells. EPO engagement activates JAK kinases and downstream PI3K/AKT signaling, promoting erythroid proliferation, differentiation, and heme synthesis. PRMT5 enhances pathway activity through multiple mechanisms, including methylation-dependent activation of PI3K regulatory subunits and suppression of the AKT/mTOR inhibitor PTEN, thereby facilitating mTORC1 signaling via TSC1/2 and Rheb. Oxidative stress–responsive inputs further modulate this axis. Dysregulation or inhibition of mTOR signaling alters erythroid maturation and is associated with changes in γ-globin expression, linking PRMT5-dependent signaling control to fetal hemoglobin regulation in SCD.

Inhibition of mTOR has been proposed as a therapeutic strategy for SCD due to its effects on oxidative stress and erythropoiesis; however, the relationship is complex, as oxidative stress can both regulate and be regulated by mTOR and GSK-3β signaling [127,128,129]. In non-erythroid systems, such as Duchenne muscular dystrophy, NADPH oxidase-derived ROS activates mTOR through the PI3K/AKT pathway, leading to suppression of autophagy [130]. mTOR activity itself is also tightly linked to erythroid maturation [131,132]. In SCD models, Wang et al. (2016) showed that the dual mTORC1/2 inhibitor INK128 improves erythrocyte count, hemoglobin concentration, and hematocrit while reducing reticulocytosis [133]. These findings support the broader hypothesis that PRMTs may influence γ-globin expression through oxidative stress–responsive PI3K/AKT/mTOR signaling pathways (Figure 2).

PI3K, along with mitogen-activated protein kinase (MAPK) and JAK-STAT signaling, plays a central role in erythroid proliferation and differentiation [134]. Engagement of the erythropoietin (EPO) receptor activates PI3K, which subsequently stimulates AKT and downstream erythroid transcription factors such as GATA-1, promoting ALAS2-mediated heme synthesis [135,136,137]. PRMT5 intersects with this network at multiple nodes: PRMT5 knockdown reduces PI3K, AKT, and mTOR signaling [138,139]; PRMT5-mediated methylation of PTEN suppresses this negative regulator of AKT/mTOR activity [140,141,142]; and PRMT5 methylates the PI3K p55 regulatory subunit, enhancing PI3K activation [143]. Together, these findings indicate extensive integration between PRMT5 and the PI3K/AKT/mTOR axis (Figure 2).

Notably, the relationship between PRMT5 and mTOR signaling is highly context-dependent (Table 1). PRMT5 inhibition suppresses mTOR signaling in CD8^+^ T cells, colorectal cancer, and bladder urothelial carcinoma models [144,145], yet enhances PI3K/AKT/mTOR pathway activity in hematopoietic stem cells, enlarges their size, and elevates protein synthesis rates [106]. Conversely, AKT/mTOR inhibition decreases PRMT5 activity in breast and lung cancer cells [146], whereas the NF-κB/mTOR/MYC axis upregulates PRMT5 in activated T-helper cells [147]. In glioblastoma, mTOR inhibition paradoxically increases PRMT5 activity, and combined inhibition of PRMT5 and mTORC1/2 exhibits synergistic antiproliferative effects [148]. These observations underscore the cell-type specificity of PRMT–mTOR crosstalk and highlight its therapeutic potential.

**Figure 3:**
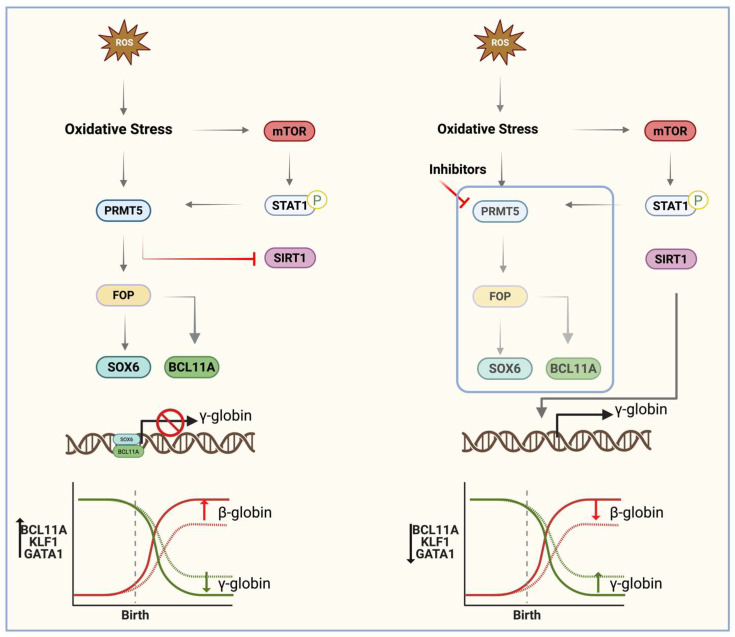
The Effects of Oxidative Stress on PRMT Activity and mTOR Signaling. Oxidative stress enhances PRMT activity and mTOR signaling via STAT1 phosphorylation, a known activator of PRMTs. This leads to suppression of Sirtuin1 (SIRT1)-mediated globin gene expression. Concurrently, FOP (Friend of PRMTs) interacts with SOX6 and BCL11A at the β-globin locus control region, where these proteins repress γ-globin expression to sustain high β-globin levels during and after hemoglobin switching. Inhibition of PRMT1/5, however, results in activation of mTOR/STAT1/SIRT1 pathway, leading to expression of γ-globin. Consequently, PRMTs represent promising therapeutic targets to alleviate sickle cell disease (SCD) severity. The second illustration proposes that inhibiting PRMTs may increase γ-globin expression, offering a potential strategy for SCD management.

In hemoglobinopathies, inhibition of mTOR signaling with rapamycin has been shown to improve clinical parameters and increase hemoglobin levels, in part through induction of γ-globin expression [133,149,150]. Although most mechanistic studies have focused on erythroid cells, mTOR signaling has also been shown to intersect with KLF1—a key transcriptional regulator of the γ-to-β globin switch—in CD4^+^ T cells, suggesting broader regulatory crosstalk that may extend to erythropoiesis [151]. In addition, Friend of PRMT (FOP), a chromatin-associated protein methylated by PRMTs, functions as a γ-globin repressor. Loss of FOP reduces SOX6 levels, thereby disrupting the SOX6–BCL11A–GATA1 repressor complex at the β-globin locus (Figure 3) [44,85].

Based on these observations, we hypothesize that mTOR influences γ-globin expression through PRMT-dependent pathways, providing a mechanistic rationale for combinatorial targeting of mTOR and PRMTs in β-hemoglobinopathies. However, dedicated studies in erythroid and sickle cell disease models will be required to validate this proposed link and determine whether these interactions operate in vivo.

### 7.2. PRMTs and ATF4 Connected Pathway

ATF4 is a stress-induced transcription factor activated in response to diverse cellular stressors, including hypoxia and oxidative stress, and is strongly upregulated in cancer and other non-erythroid disease models [152,153]. In these contexts, ATF4 functions as a master regulator of the integrated stress response (ISR), serving as a common downstream effector for multiple signaling pathways, including those converging on mTOR [152]. Indeed, studies in non-erythroid systems demonstrate that ATF4 can enhance mTOR pathway activity through regulation of autophagy and induction of adaptive stress-response genes [154]. In addition, under oxidative stress conditions, PRMT1—again studied primarily outside the erythroid lineage—methylates ATF4 at Arg239 via recruitment by B-cell translocation gene 1 (BTG1), thereby promoting transcription of pro-apoptotic genes in cancer cells [153].

By contrast, a distinct body of work has examined ATF4 function directly in erythroid systems and β-globinopathies. In β-globin knockout erythroid cells, ATF4 activity is significantly reduced, resulting in downregulation of multiple canonical ATF4 target genes. Conversely, mutation of endogenous ATF4 increases γ-globin expression, indicating a functional link between ATF4 activity and the fetal-to-adult globin switch. Notably, although BCL11A levels are reduced in both HBB-deficient and ATF4-mutant erythroid cells, ChIP-seq analyses reveal that ATF4 does not bind the BCL11A locus directly. Instead, ATF4 occupies the HBS1L–MYB intergenic enhancer region. Because MYB is a known regulator of fetal hemoglobin and promotes BCL11A expression [80,155], and because MYB cooperates with KLF1 in erythroid transcriptional programs, these findings support a model in which ATF4 indirectly regulates γ-globin expression through modulation of the MYB–KLF1–BCL11A axis. Under β-globin-associated cellular stress, reduced ATF4 activity may disrupt this regulatory network, thereby contributing to γ-globin induction—an effect with clear therapeutic relevance for β-hemoglobinopathies. However, while PRMT1-mediated methylation of ATF4 is mechanistically informative, whether this modification directly contributes to γ-globin regulation in erythroid cells remains to be determined.

### 7.3. PRMTs and JAK-STAT Pathway

**Figure 4:**
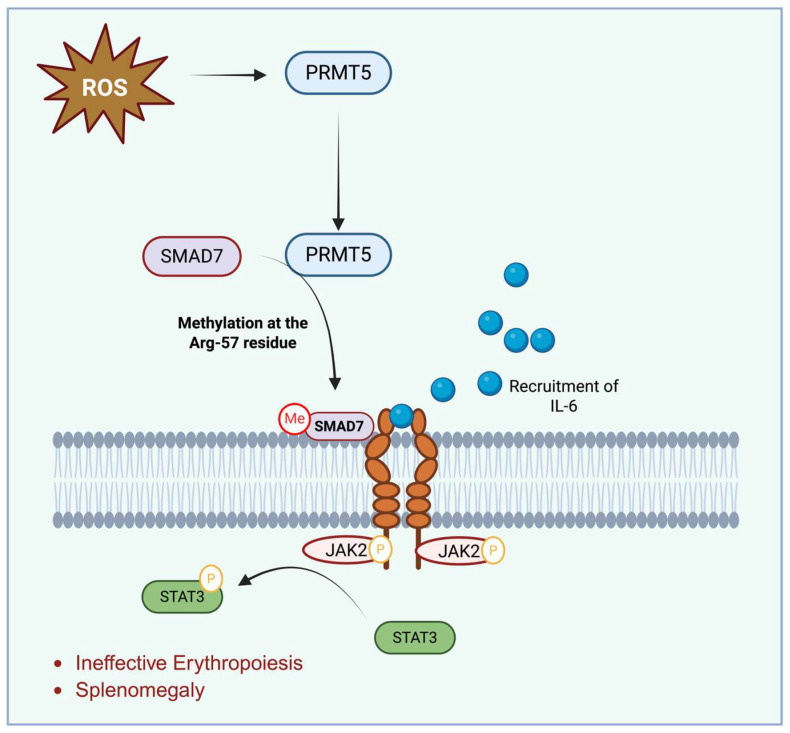
The Role of Oxidative Stress in Promoting Interaction of PRMTs with Smad7. Oxidative stress promotes the interaction of cellular PRMTs with Smad7, leading to its methylation at the Arg-57 residue. Methylated SMAD7 binds to gp130 and recruits IL-6, initiating downstream signaling. Upon phosphorylation, gp130 activates Jak2, which in turn phosphorylates STAT3—a transcription factor implicated in inflammation and splenomegaly. Inhibition of PRMT5 or Jak2 reduces STAT3 phosphorylation, thereby attenuating spleen enlargement and inflammation.

The IL-6/JAK/STAT3 pathway plays a critical role in cancer and inflammatory diseases and is also activated under anemic and hypoxic stress conditions. In β-thalassemia, hypoxia-driven increases in IL-6 and erythropoietin (EPO) contribute to heightened JAK/STAT signaling, expansion of erythroid progenitors, and splenic extramedullary hematopoiesis [17,156]. Clinical and preclinical studies evaluating JAK2 inhibitors have demonstrated improvements in ineffective erythropoiesis and reductions in splenomegaly [157], highlighting the relevance of this pathway in hematologic disorders. Although PRMT5 was originally identified as a JAK2-binding protein (Table 1), its precise role in JAK/STAT regulation remained unclear until studies in cancer cell models revealed that PRMT5 is essential for IL-6-mediated STAT3 activation. Cai et al. (2021) demonstrated that PRMT5 knockdown or pharmacologic inhibition suppresses STAT3 phosphorylation and IL-6 responsiveness (Table 1) [158]. These studies further showed that PRMT5 methylates SMAD7 at Arg-57, promoting SMAD7’s interaction with the IL-6 co-receptor gp130 and enabling robust STAT3 activation (Figure 4) [158].

To date, there is no direct evidence that PRMT5 modulates JAK/STAT signaling through SMAD7 methylation in erythroid progenitors, nor that this mechanism contributes to ineffective erythropoiesis in SCD. Additional mechanistic work is needed to determine whether PRMT5 or other PRMT isoforms engage similar regulatory pathways in erythroid cells under oxidative stress, anemia-induced cytokine signaling, or SCD-associated inflammatory environments.

### 7.4. PRMTs and SIRT1

SIRT1 is a nicotinamide adenosine dinucleotide (NAD)-dependent deacetylase encoded by the SIRT1 gene that removes acetyl groups from histone and non-histone proteins and thereby regulates diverse physiological and transcriptional programs [159,160,161]. Through deacetylation of multiple transcription factors and co-regulators, SIRT1 modulates chromatin accessibility, transcription complex assembly, and gene expression patterns [161,162,163].

In erythroid cells, SIRT1’s histone deacetylase activity has direct implications for γ-globin regulation. SIRT1 has been shown to induce HBG expression by promoting chromatin conformational changes that facilitate looping between the LCR and HBG promoter. This mechanism involves deacetylation of H4K16Ac and enhanced recruitment of RNA polymerase II at the γ-globin promoter. In addition, SIRT1 also decreases expression of key γ-globin repressors, including BCL11A, KLF1, HDAC1, and HDAC2, establishing SIRT1 as a positive regulator of HbF within the erythroid lineage [163]. By contrast, studies linking PRMTs to SIRT1 regulation derive from non-erythroid systems, most notably retinal pigment epithelial cells [164]. In these models, PRMT1 and PRMT4 (CARM1) upregulate SIRT1 expression under oxidative stress and reduce apoptosis [164]. While these findings offer mechanistic insight into how PRMTs may modulate SIRT1 under oxidative conditions, they have not yet been demonstrated in erythroid cells. Taken together, these observations suggest that enhancing SIRT1 activity could promote γ-globin expression and potentially ameliorate SCD pathology. However, because PRMT-dependent regulation of SIRT1 has not been validated in erythroid or SCD models, further studies are required to determine whether PRMT1- or PRMT4-mediated SIRT1 induction occurs in erythroid progenitors and contributes to HbF regulation in vivo.

### 7.5. PRMTs and FOXO3 Connected Pathway

**Figure 5:**
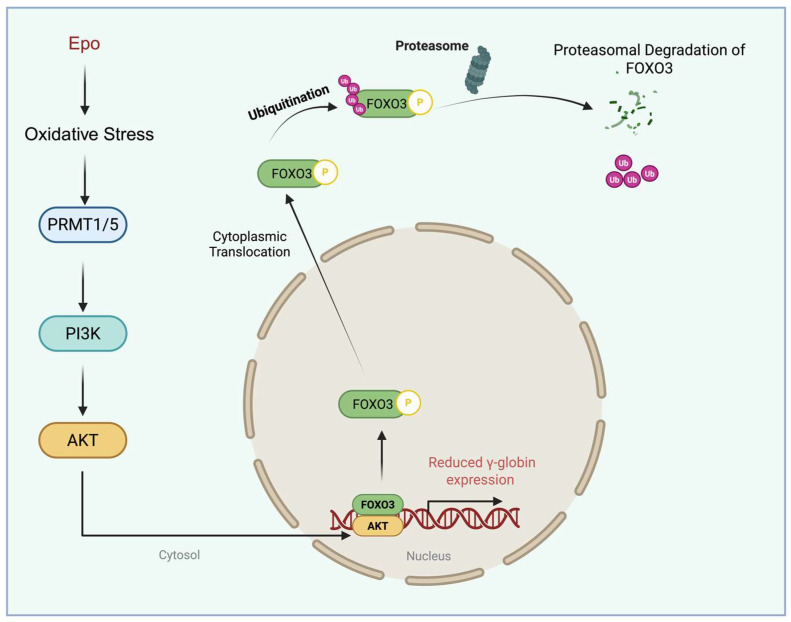
FOXO3 Regulation of Cell Proliferation and γ-Globin Gene Expression via the PRMT-activated PI3K/AKT pathway. Under basal conditions, nuclear FOXO3 promotes cell proliferation and γ-globin expression. During erythropoietin-induced oxidative stress, FOXO3 is phosphorylated by AKT through the PRMT-activated PI3K/AKT pathway. This leads to FOXO3 translocation to the cytosol, ubiquitination, and proteasomal degradation. Loss of nuclear FOXO3 results in reduced γ-globin expression and induction of autophagy.

Forkhead box O (FOXO)3 is a transcription factor involved in multiple biological processes, including antioxidant defense, longevity, cell-cycle control, γ-globin regulation, and erythroid maturation [165,166,167,168,169]. Evidence from erythroid models strongly supports a role for FOXO3 in hemoglobin regulation: using ex vivo differentiation of human CD34^+^ cells, FOXO3 knockdown was shown to reduce γ-globin expression without affecting β-globin, identifying FOXO3 as a positive regulator of γ-globin acting independently of erythroid maturation kinetics [170] (Figure 5). These data position FOXO3 as a promising therapeutic target for sickle cell disease (SCD) and β-thalassemia, particularly for individuals who respond poorly to hydroxyurea. In vivo models further underscore FOXO3’s physiological importance. FOXO3-deficient animals exhibit rapid mortality following oxidative stress due to impaired activation of antioxidant enzymes [168], demonstrating FOXO3’s essential role in redox homeostasis.

In erythroid progenitors, FOXO3 function is tightly controlled by the PI3K/AKT pathway. Upon EPO stimulation, AKT phosphorylates FOXO3 at conserved residues, promoting its interaction with 14-3-3 proteins and driving its export from the nucleus [171,172]. This cytosolic sequestration leads to ubiquitination and proteasomal degradation, thereby suppressing FOXO3’s transcriptional activity [171,172,173]. Because nuclear FOXO3 promotes γ-globin expression, dysregulated PI3K/AKT signaling in erythroid cells—particularly under oxidative stress—can diminish γ-globin production.

PRMTs also contribute to FOXO3 regulation. PRMT1 indirectly enhances FOXO3 activity by suppressing PRMT6, which methylates and activates FOXO3; thus, PRMT1 depletion increases nuclear localization of FOXO1/3 [174]. These findings indicate a nuanced interplay among PRMT family members in supporting FOXO-dependent transcription. Several mechanisms describing PRMT-mediated FOXO3 regulation arise from non-erythroid models, including skeletal muscle and stress-response pathways. In these systems, elevated PRMT1 or PRMT5 activity under oxidative stress reduces FOXO3 nuclear localization via enhanced PI3K/AKT signaling, contributing to autophagy induction, mitotic arrest, and impaired proliferation [174,175] (Figure 5). While informative, these PRMT1/PRMT5–FOXO3 interactions have not yet been experimentally validated in erythroid or SCD models.

Under basal conditions, nuclear FOXO3 supports cell proliferation and γ-globin expression. During EPO-induced oxidative stress, PRMT-dependent activation of the PI3K/AKT pathway promotes FOXO3 phosphorylation, cytosolic translocation, and degradation. The resulting loss of nuclear FOXO3 reduces γ-globin transcription and triggers autophagy. Consequently, modulating PRMT1 and PRMT5 activity may offer therapeutic benefit for ineffective erythropoiesis and impaired HbF induction in β-thalassemia and SCD, although erythroid-specific validation remains necessary.

### 7.6. PRMTs and Nrf2-Keap1 Pathway

Numerous antioxidant systems protect cells from excessive ROS, and a central regulator of this defense network is the Nrf2/Keap1 pathway. Under basal conditions, Nrf2 is sequestered in the cytoplasm by Keap1, which targets it for ubiquitin-mediated degradation [176,177,178]. During oxidative stress, the Nrf2–Keap1 complex dissociates, allowing Nrf2 to accumulate in the nucleus, where it heterodimerizes with small musculoaponeurotic fibrosarcoma (sMaf) proteins. These Nrf2–sMaf complexes bind antioxidant response elements (AREs) to initiate transcription of cytoprotective genes [179]. Transcriptional activation by Nrf2 is further modulated by epigenetic co-regulators. Acetylation of Nrf2 by the p300/CBP complex increases its dissociation from Keap1 and enhances its DNA-binding affinity [180]. Notably, p300/CBP complexes contain PRMT1 and CARM1 as coactivators, which together amplify Nrf2-dependent transcriptional responses [103,181,182,183,184]. In addition to its coactivator role, PRMT1 directly methylates Nrf2 at arginine 437, strengthening both its DNA-binding and transactivation capabilities [179]. These regulatory layers reinforce Nrf2 function during oxidative stress and illustrate how PRMTs can integrate into redox-responsive signaling networks.

**Figure 6:**
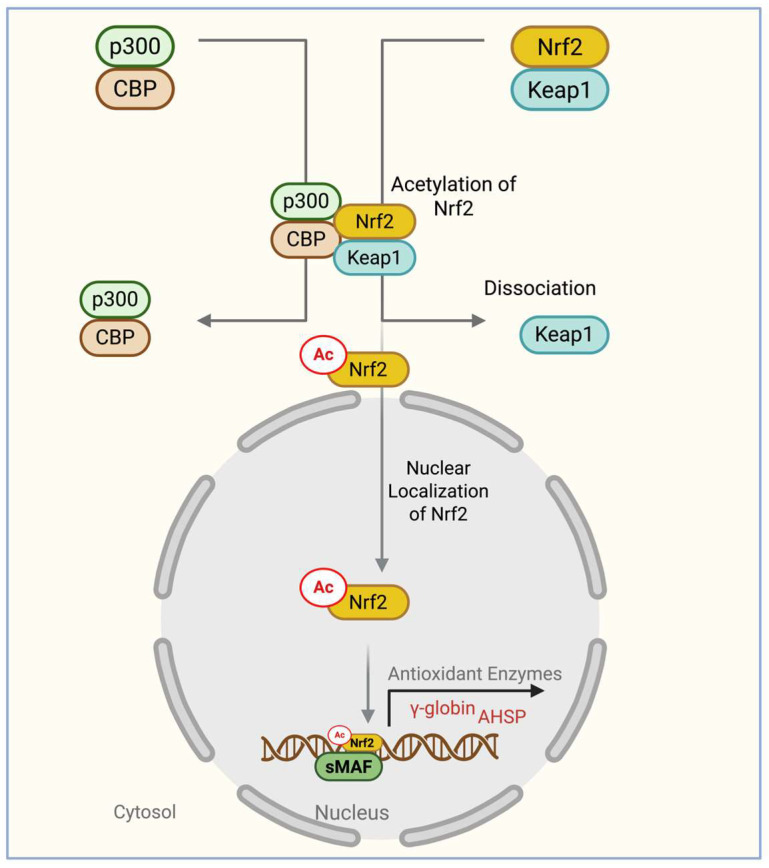
p300/CBP-Mediated Activation of Nrf2 and Induction of Antioxidant Gene Expression. The p300/CBP complex, which includes PRMT1 and CARM1, acetylates Nrf2, leading to its dissociation from Keap1. Free Nrf2 translocates to the nucleus and forms a heterodimer with sMAF. This complex binds to antioxidant response elements (AREs), inducing expression of antioxidant enzymes including γ-globin and alpha-hemoglobin-stabilizing protein (AHSP).

Activated Nrf2–sMaf heterodimers induce transcription of several antioxidant and cytoprotective genes, including heme oxygenase-1 (HO-1). HO-1 catalyzes the degradation of heme into biliverdin, free iron, and carbon monoxide (CO). Importantly, CO has been shown to help maintain the biconcave shape and membrane flexibility of sickle erythrocytes in SCD models, thereby mitigating downstream hemolytic and vaso-occlusive complications [178,185]. Beyond antioxidant defense, Nrf2–sMaf complexes also regulate erythroid-specific targets relevant to hemoglobin homeostasis. They enhance the expression of γ-globin as well as alpha-hemoglobin-stabilizing protein (AHSP), which binds free α-globin to prevent its precipitation and associated oxidative damage [186,187]. Through these combined effects—boosting HbF production and stabilizing α-globin—Nrf2-driven transcription helps alleviate oxidative stress in erythroid cells and supports more effective erythropoiesis (Figure 6).

## 8. Potential Therapeutic Targeting of PRMTs in SCD

The emerging roles of PRMTs in regulating the cellular stress response and, specifically, in modulating γ-globin gene expression position these enzymes as compelling therapeutic targets for SCD [178]. The rationale for PRMT-directed therapy is twofold: (1) to directly counteract oxidative stress-driven injury in RBCs, and **(2)** to indirectly ameliorate SCD pathophysiology by inducing HbF, a well-established modifier of disease severity [85].

The therapeutic promise of this approach is supported by rapid advances in the development of PRMT inhibitors for oncology. Medicinal chemistry efforts have yielded a diverse array of selective small-molecule inhibitors targeting individual PRMT isoforms. Notably, PRMT5 inhibitors have progressed into clinical trials for multiple cancers, reflecting their effectiveness in disrupting epigenetic programs that sustain tumor growth [188,189,190]. These clinical advances suggest that repurposing PRMT inhibitors—or designing hematology-specific analogs—may be a feasible and strategic avenue for SCD therapy.

Targeting PRMT5 offers a mechanistic opportunity to relieve transcriptional repression of the γ-globin gene. Pharmacological inhibition of PRMT5 could diminish the deposition of repressive histone marks and thereby reactivate γ-globin expression. Such HbF induction is a validated therapeutic goal in SCD, as demonstrated by the clinical success of hydroxyurea [98,101,104]. Complementing this transcriptional approach, inhibition of PRMT1 has been shown to increase γ-globin at the translational level, revealing an additional layer of HbF regulation. Thus, dual targeting of PRMT5 (transcriptional repression) and PRMT1 (translational suppression) may produce synergistic HbF induction in β-hemoglobinopathies.

Beyond PRMT1 and PRMT5, growing attention has turned to PRMT7, whose activity intersects with cellular stress pathways. PRMT7 inhibition may provide a dual benefit in SCD by both sensitizing erythroid precursors to stress-responsive γ-globin induction and potentially protecting RBCs from oxidative injury. The recent development of selective PRMT7 inhibitors [191] highlights the expanding interest in this enzyme and underscores the need for further investigation into PRMT7-dependent mechanisms within erythropoiesis.

Collectively, these emerging insights establish PRMT inhibition as a promising and multifaceted therapeutic strategy for SCD—capable of mitigating oxidative damage, reactivating HbF expression, and targeting fundamental epigenetic and post-transcriptional pathways involved in globin gene regulation.

## 9. Conclusions

The induction of HbF; α2γ2 in RBCs represents a powerful therapeutic approach for SCD. By increasing HbF levels, the intracellular concentration of HbS; α_2_β^s^_2_ is reduced, thereby inhibiting its pathological polymerization and the downstream cascade of vaso-occlusive and hemolytic events. However, in the absence of effective γ-globin inducers, maturing erythroid cells accumulate predominantly adult hemoglobin and become highly vulnerable to oxidative injury. This heightened susceptibility reflects the substantial oxidative burden characteristic of SCD, which activates a wide array of cellular signaling pathways—including several governed by PRMTs.

This review synthesizes the emerging and increasingly recognized roles of PRMT family members in erythropoiesis, with particular attention to potential connections between PRMT activity and oxidative stress–responsive pathways in erythroid cells. Although many mechanistic details remain to be validated in erythroid systems, PRMTs have been implicated in several major regulatory networks—including PI3K/AKT, mTOR, Nrf2–Keap1, ATF4-mediated stress signaling, SIRT1 activation, and FOXO3 transcriptional control—that respond to redox imbalance. Through these intersecting pathways, PRMTs may influence antioxidant defenses, hemoglobin switching, erythroid maturation, and erythroblast survival under redox-challenged conditions. Together, these observations suggest that PRMT-dependent signaling could couple redox sensing to epigenetic control of erythroid gene programs, including globin regulation.

In summary, accumulating evidence positions PRMTs as central epigenetic modulators at the interface of oxidative stress and globin gene regulation. The working hypothesis that reactive oxygen species modulate PRMT activity in erythroid cells—thereby influencing the developmental transition from fetal to adult hemoglobin—offers a framework for understanding HbF control. This paradigm opens avenues for therapeutic intervention in sickle cell disease, with the potential to target PRMT-dependent pathways to both mitigate oxidative damage and enhance HbF production.

## 10. Future Directions and Therapeutic Implications

The growing evidence implicating PRMTs in γ-globin regulation highlights several important avenues for future investigation. First, it will be essential to define which PRMT isoforms—particularly PRMT1, PRMT5, and PRMT7—play the most critical roles in oxidative-stress-mediated control of γ-globin expression. Clarifying these isoform-specific functions will help delineate how redox cues are integrated into the epigenetic networks governing hemoglobin switching. Second, the efficacy and safety of selective PRMT inhibition must be rigorously evaluated in models of human erythropoiesis and SCD. These studies should also assess potential synergistic effects between PRMT inhibitors and established HbF-inducing agents such as hydroxyurea. The interplay between PRMT activity and other epigenetic regulators—including DNA methyltransferases and histone deacetylases—represents an additional layer of regulation that warrants systematic exploration.

From a translational perspective, the development of PRMT-based therapeutic strategies will require careful balancing of HbF induction with preservation of healthy hematopoiesis. Given the essential roles of PRMT1 and PRMT5 in erythroid biology, approaches employing transient, partial, or cyclical inhibition may offer a means to enhance γ-globin expression while minimizing hematologic toxicity. Interindividual differences, including genetic modifiers that influence HbF levels, may further shape patient responses to PRMT-targeted therapies.

Finally, comprehensive profiling of the transcriptional and epigenomic consequences of PRMT inhibition will be critical for understanding potential off-target effects and for guiding the translation of preclinical findings into clinically viable strategies. Pursuing these lines of inquiry holds significant promise for advancing mechanism-based therapies that address the fundamental pathophysiology of hemoglobinopathies—and sickle cell disease in particular.

## Data Availability

No new data were created or analyzed in this study. Data sharing is not applicable to this article.
